# Glycated Proteins, Glycine, Acetate, and Monounsaturated Fatty Acids May Act as New Biomarkers to Predict the Progression of Type 2 Diabetes: Secondary Analyses of a Randomized Controlled Trial

**DOI:** 10.3390/nu14235165

**Published:** 2022-12-05

**Authors:** Francisco Canet, Jacob J. Christensen, Victor M. Victor, Kristin S. Hustad, Inger Ottestad, Amanda Rundblad, Thomas Sæther, Knut Tomas Dalen, Stine M. Ulven, Kirsten B. Holven, Vibeke H. Telle-Hansen

**Affiliations:** 1 Service of Endocrinology and Nutrition, University Hospital Doctor Peset, Foundation for the Promotion of Health and Biomedical Research in the Valencian Region (FISABIO), 40617 Valencia, Spain; 2 Department of Nutrition, Institute of Basic Medical Sciences, Faculty of Medicine, University of Oslo, 0317 Oslo, Norway; 3 Department of Physiology, School of Medicine, University of Valencia, Av Blasco Ibáñez 13, 46010 Valencia, Spain; 4 Department of Molecular Medicine, Institute of Basic Medical Sciences, University of Oslo, 0317 Oslo, Norway; 5 Norwegian National Advisory Unit on Familial Hypercholesterolemia, Oslo University Hospital Rikshospitalet, 0424 Oslo, Norway; 6 Department of Nursing and Health Promotion, Faculty of Health Sciences, Oslo Metropolitan University, 0130 Oslo, Norway

**Keywords:** diabetes, salmon fish protein, fishmeal, metabolome, transcriptome, metabolic profile

## Abstract

Food protein or food-derived peptides may regulate blood glucose levels; however, studies have shown inconsistent results. The aim of the present study was to characterize subgroups of individuals with increased risk of type 2 diabetes (T2D) and to investigate the cardiometabolic effects of fish protein in the same subgroups. We first divided participants into high insulin_iAUC_ and low insulin_iAUC_ subjects based on their insulin incremental area under the curve (iAUC) levels after a 2 h oral glucose tolerance test (OGTT), and secondly based on whether they had received 5.2 g salmon fish protein or placebo for 8 weeks, in a previously conducted randomized controlled trial (RCT). We then profiled these groups by analyzing plasma metabolomics and peripheral blood mononuclear cell (PBMC) gene expression. Compared to the low insulin_iAUC_ group, the high insulin_iAUC_ group had higher plasma concentrations of monounsaturated fatty acids (MUFAs) and glycated proteins (GlycA) and lower concentrations of glycine and acetate. After intervention with fish protein compared to placebo, however, only acetate was significantly increased in the low insulin_iAUC_ group. In conclusion, we identified metabolic biomarkers known to be associated with T2D; also, intervention with fish protein did not affect cardiometabolic risk markers in subgroups with increased risk of T2D.

## 1. Introduction

Type 2 diabetes (T2D) is estimated to affect more than 425 million individuals globally [1]. Impaired insulin secretion due to dysfunctional pancreatic beta-cells and/or peripheral insulin resistance is established characteristics of T2D, giving dysregulated metabolism of carbohydrates, fatty acids, and protein [2]. Prediabetes is an early stage of this continuum and is a high-risk condition for developing T2D [2]. The term prediabetes is increasingly being used to refer to individuals who are at increased risk of developing T2D [3]. Individuals with prediabetes have elevated levels of fasting serum insulin and Homeostatic Model Assessment for Insulin Resistance (HOMA-IR), indicating a direct relationship between prediabetes and insulin resistance [4]. However, individuals with prediabetes are a diverse population in terms of pathophysiology and clinical presentation [2] and may display variations in fasting or postprandial blood glucose concentrations [4]. Excess adipose tissue increases insulin resistance [5], and the prevalence of prediabetes gradually increases with increasing body mass index (BMI) and waist circumference [4]. Moreover, prediabetes alters serum lipid profiles (increased triglycerides and decreased high-density lipoprotein (HDL)) and increases cardiac risk ratios (total-cholesterol/HDL-C and low-density lipoprotein cholesterol (LDL-C)/HDL-C) and resting blood pressure. Thus, prediabetes is also an important predictor of metabolic syndrome [4]. Individuals with prediabetes and/or metabolic syndrome are at high risk of developing T2D and cardiovascular diseases [4].

The ability to predict progression from impaired glucose tolerance to T2D is, therefore, highly valuable for improved prevention. However, identifying those at the highest risk remains challenging. Some new biomarkers for cardiovascular and T2D risk have been suggested, including lipoprotein insulin resistance (LP-IR) score [6,7,8], and circulating glycoprotein acetyls (GlycA) [6,9] branched-chain amino acids (BCAAs) [6] and glycine [6]. Furthermore, fatty acids are important signaling molecules [10,11], and circulating levels of several fatty acids are increased with insulin resistance [12]. Increased plasma levels of monounsaturated fatty acids (MUFAs) are associated with cardiovascular risk [13] and with increased HbA1c and fasting glucose levels [14]. The effect of fatty acids on diabetic risk is suggested to be through inflammatory mechanisms [15], oxidative stress [16], and hepatic de novo lipogenesis [17]. However, prediabetes is still sub-optimally characterized with respect to a broad risk assessment, and whether these newly suggested biomarkers are more sensitive to dietary intervention in people with increased risk of T2D is still to be elucidated.

There is conclusive evidence from randomized controlled trials (RCT) that intensive lifestyle interventions (diet and/or exercise) [2,18] and medications (such as metformin, alpha-glucosidase inhibitors, and thiazolidinediones) can delay or prevent the onset of T2D in high-risk individuals [2]. Observational follow-up of RCT participants has shown that the beneficial effects of lifestyle interventions may persist over time, improve quality of life, and are both safe and cost-effective [2,19]. A healthy diet is important for both the prevention and management of T2D [20]. Food protein or food-derived peptides have been found to regulate blood glucose levels [21,22,23,24]. Although interventions with fish proteins have shown promising effects on cardiometabolic risk markers [25,26,27,28,29] and glycemic regulation in human studies [28,30], these observations are inconsistent [26,31,32,33]. In a recent RCT, Hustad et al. investigated the effect of an 8-week intervention with salmon fish protein on cardiometabolic risk markers in people with increased risk of T2D [34]. They found that intervention with salmon fish protein had no effect on glycemic regulation in individuals with an increased risk of T2D [34]. However, the participants in the study had large variability in plasma glucose and insulin values. Hence, the lack of a group effect on glycemic regulation could be due to the heterogeneous nature of prediabetes in the studied individuals.

The aim of the present study was to characterize subgroups of individuals with increased risk of T2D who participated in the previous intervention study by Hustad et al. [34]. Participants were grouped based on their insulin incremental area under the curve (iAUC) levels after a 2 h oral glucose tolerance test (OGTT). Metabolome and transcriptome analyses were applied to measure alterations in cardiometabolic risk markers after intake of salmon fish protein in subgroups with low insulin_iAUC_ and high insulin_iAUC_.

## 2. Materials and Methods

### 2.1. Subjects

In the present study, we used clinical, metabolome, and transcriptome data from the FishMeal study [34]. The FishMeal study was a human RCT investigating the effects of salmon fish protein intake on cardiometabolic risk markers. The inclusion criteria were non-diabetic men and women ≥20 years and elevated blood glucose defined as either fasting serum (s)-glucose ≥5.6 mmol/L, 2 h OGTT-s-glucose ≥6.5 mmol/L or HbA1c ≥40 mmol/mol (≥5.8%). People with diabetes were excluded from the study. Further description of the exclusion criteria is available in Hustad et al. [34]. The participants were included in an 8-week double-blind, randomized controlled parallel study with 5.2 g of salmon fish protein (corresponding to 7.5 g of fishmeal) compared with a placebo, as previously described. The participants were instructed to limit their fish and seafood intake to a maximum of 150 g per week and otherwise maintain their usual lifestyle habits throughout the study. The salmon fishmeal contained 69.7 g of protein and 13.2 g of fat/100 g. A more detailed description of the content can be found in [34].

The FishMeal human intervention study analyzed data from 74 participants. For the present study, two participants were excluded due to a lack of data on fasting or 2 h insulin concentrations, resulting in a study sample of 72 participants.

### 2.2. Blood Sampling and Biochemical Routine Measures

Venous blood samples were drawn after an overnight fast (≥10 h). Serum was obtained from silica gel tubes (Becton, Dickinson, and Company, San Jose, CA, USA) and kept at room temperature for 30–60 min until centrifugation (1500× *g*, 15 min). Plasma was obtained from K2EDTA tubes (Becton, Dickinson, and Company), immediately placed on ice, and centrifuged within 10 min (2000× *g*, 4 °C, 15 min).

Serum concentrations of fasting glucose, insulin, HbA1c, triglycerides, total cholesterol, LDL-cholesterol, HDL-cholesterol, high-sensitive C-reactive protein (hsCRP), and glucose and insulin after a 2 h OGTT (75 g glucose load) were measured by standard methods at an accredited routine laboratory (Fürst Medical Laboratory, Oslo, Norway).

### 2.3. NMR Spectroscopy

Circulating metabolites, before and after the intervention, were quantified at a high throughput proton NMR metabolomics facility (Nightingale Health Ltd., Helsinki, Finland), giving a snapshot of systemic metabolism [35]. The method measures 250 metabolites (lipoprotein subclasses, fatty acids, glycolysis-related metabolites, amino acids, ketone bodies, fluid balance markers, and inflammatory markers) in fasting EDTA plasma.

### 2.4. Isolation of Peripheral Blood Mononuclear Cells and RNA

Peripheral blood mononuclear cells (PBMCs) from blood samples drawn at fasting, before and after the intervention, were isolated with BD Vacutainer Cell Preparation tubes (Becton, Dickinson). The method is well-documented for PBMCs isolation with more than 90% purity. PBMCs were isolated according to the manufacturer’s instructions and stored at −80 °C until RNA isolation. Total RNA was isolated using the RNeasy kit with QIAshredder homogenization of the cell lysates and DNase treatment using the automated protocol for the QIAcube according to the manufacturer’s instructions (Qiagen, Valencia, CA, USA). The quantity and quality of the isolated RNA were analyzed with the NanoDrop ND-1000 Spectrophotometer (Thermo Fisher Scientific, Gothenburg, Sweden) and the Agilent RNA 6000 Nano kit using Agilent 2100 Bioanalyzer (Agilent Technologies, Santa Clara, CA, USA), respectively. Six participants were excluded due to missing PBMCs and hence RNA, leaving 66 participants for the gene expression analyses.

### 2.5. Nanostring Gene Expression Assay

RNA expression analysis was performed using the NanoString nCounter system and the nCounter Metabolic Pathways Panel (Nanostring Technologies, https://nanostring.com/support-documents/metabolic-pathways-panel-gene-list/, accessed on 14 October 2022). This panel contains code sets that cover 768 mRNAs annotated to different metabolic pathways, including 20 reference genes, as well as positive and negative controls. All gene name abbreviations are listed in Supplementary Table S1. In addition, the panel was customized by adding 30 additional code sets covering immune and inflammation response and lipid metabolism-related mRNAs (Supplementary Table S2). The procedure was performed according to the manufacturer’s instructions for nCounter Panel-Plus with an RNA input of 75 ng per sample.

Samples with an imaging quality control >75%, binding density between 0.1 and 1.8, positive control linearity >0.95, and the lowest positive control (0.5 fm) count higher than 2 SD above the negative controls were included. Samples were normalized to remove both technical and biological variation. The background threshold was set to be the geometric mean of the negative controls. Each sample was normalized to the mean of its positive controls relative to the geometric mean of positive controls in all samples. Finally, all samples were normalized to all 20 reference genes included in the panel (ABCF1, FCF1, SAP130, MRPS5, COG7, TBP, USP39, EDC3, DHX16, UBB, NRDE2, TLK2, DNAJC14, POLR2A, SDHA, G6PD, OAZ1, TBC1D10B, STK11IP, and AGK). The quality control and the technical and biological normalization were performed in the nSolver analysis software version 4.0 (NanoString Technologies). The stability of the 20 reference genes was assessed using NormFinder.

### 2.6. Statistics and Bioinformatics Analyses

#### 2.6.1. Tools

All analyses were performed in R version 4.1.2 and RStudio IDE (https://www.rstudio.com/, accessed on 2 December 2021). In the following sections, we indicate the packages and specific functions that were relevant in the following format: *package*::*function (setting)*. We used tidyverse tools for data management and visualization [36].

#### 2.6.2. Linear Regression Models

We used the R/Bioconductor software package *limma* [37] for running the linear models and assessing differential metabolite concentrations and gene expressions. We adjusted for age, sex, and BMI in the linear models for the metabolomics data and the gene expression data. We also included smoking as a covariate in the gene expression analyses.

#### 2.6.3. Pre-Processing

We pre-processed the data to optimize downstream modeling. Metabolite concentrations were log-transformed (*base::log1p*), centered, and scaled (*base::scale*). Centering and scaling were conducted to make metabolites directly comparable in the same downstream analysis and forest plot visualization. For the intervention data, we centered and scaled independently the before and after intervention values and then calculated the difference in metabolites concentration. Gene expression data were log transformed (*base::log2*).

#### 2.6.4. Stratification of the Participants and Metabolome and Transcriptome Characterization before Intervention

Before the intervention, the participants in the FishMeal study were divided into three groups based on their insulin incremental area under the curve (iAUC) levels after a 2 h OGTT. We calculated the insulin iAUC using two insulin measurements (fasting and 2 h insulin serum concentration) and the trapezoid method (*DescTools::AUC* [38]). Participants in the lowest tertile (below 33.3%) were classified into the low insulin_iAUC_ group (*n* = 24), whereas those in the highest tertile (above 66.6%) to the high insulin_iAUC_ group (*n* = 24). The two groups were characterized and compared for metabolic profiling (including metabolites and PBMC gene expression profiles). We included all three groups in the linear modeling and focused on the comparison between the low and high insulin_iAUC_ groups. The linear model of the group characterizations before intervention (*limma::lmFit* followed by *limma::eBayes*) was defined as: metabolite or gene = insulin_iAUC_ group + covariates. We considered that metabolites concentrations and gene transcripts expression with *p* < 0.05 (no FDR correction) were different between the low and high insulin_iAUC_ groups. Supplementary Figure S1 illustrates the outline of the analysis pipeline for the characterization of the subgroups before intervention.

#### 2.6.5. Analyses of Metabolic and Gene Expression Profiles in the Subgroups after Intervention

The same metabolites and gene transcripts were measured after intervention with fish protein or placebo in the low insulin_iAUC_ groups (*n* = 16 and *n* = 8, respectively) and the high insulin_iAUC_ groups (*n* = 12 and *n* = 12, respectively). We assessed changes in plasma metabolite concentrations and changes in PBMC gene expression levels before and after intervention in the low and high insulin_iAUC_ groups.

We defined the linear model (*limma::lmFit* followed by *limma::eBayes*) for the effects of the fish protein intervention as changes in metabolite or gene =insulin_iAUC_ group + treatment group + covariates. Then, we computed the estimated coefficient for the contrasts between fish protein-treated low insulin_iAUC_ vs. placebo-treated low insulin_iAUC_ and fish protein-treated high insulin_iAUC_ vs. placebo-treated high insulin_iAUC_. Metabolite concentrations and gene transcript expressions with *p* < 0.05 (no FDR correction) were considered altered when compared to placebo. Supplementary Figure S2 illustrates the outline of the analysis pipeline of the fish protein intervention.

#### 2.6.6. Gene Set Enrichment Analysis and Competitive Gene Set Testing

We used the list of genes (obtained with *limma::lmFit* followed by *limma::eBayes*) (*p* values < 0.05, no FDR correction) and searched for “enriched pathways” in order to understand patterns in gene expression that could be different between the low insulin_iAUC_ and high insulin_iAUC_ groups and affected by the fish protein intervention. This consisted of testing if the obtained list of genes over-represented Gene Ontology (GO) terms [39,40] or KEGG pathways [41] more than expected by chance. The lists of genes were tested with the *limma::kegga* function for KEGG pathways and the *limma::goana* function for GO terms. We restricted the universe of genes only to genes found in our nCounter panel (Supplementary Tables S1 and S2). The *p*-values returned by *limma::goana* and *limma::kegga* functions are unadjusted for multiple testing because GO terms and KEGG pathways are often overlapping, and standard methods of *p*-value adjustment may be very conservative [42].

To broaden our search, we used the Hallmark gene sets from the Molecular Signatures Database (MSigDB) [43]. The Hallmarks gene sets summarize and represent specific biological states or processes and display coherent expression [43]. With the Hallmark gene sets, we performed competitive gene set testing, which allowed us to distinguish the most important biological process from those that are less important. For this analysis, we used the Hallmark gene set and the Camera method (*limma::camera*) [44]. Because the Camera method also considers fold changes, we could interpret if the biological pathways were up- or downregulated.

## 3. Results

### 3.1. Subgroup Characterization before Intervention

For this exploratory study, 72 individuals with a high risk of T2D (*n* = 27 males/45 females) were divided into three groups (tertiles) based on their insulin iAUC values before intervention. The groups with the lowest and the highest insulin_iAUC_ were compared.

Table 1 shows the anthropometric and biochemical characteristics of the study population before intervention. The median age was 53.0 years in the low insulin_iAUC_ group and 61.5 years in the high insulin_iAUC_ group. The two groups had similar BMI (median BMI in the low insulin_iAUC_ group: 32.4 kg/m^2^ and high insulin_iAUC_ group: 33.0 kg/m^2^). The proportion of women to men was higher in both groups (low insulin_iAUC_ group: 71% and high insulin_iAUC_ group: 63%). The proportion of participants that used tobacco daily was higher in the high insulin_iAUC_ group (26.3%) compared to the low insulin_iAUC_ group (9.1%). We found differences in parameters related to glucose metabolism and insulin sensitivity (glycemic regulation) between the low insulin_iAUC_ and high insulin_iAUC_ groups as expected, in which the low insulin_iAUC_ group being the one with the lowest glucose 2 h, fasting insulin, insulin 2 h, and HOMA-IR and with the highest Matsuda index (Table 1).

### 3.2. Subgroup Differences in Metabolic Profile before Intervention

**Figure 1:**
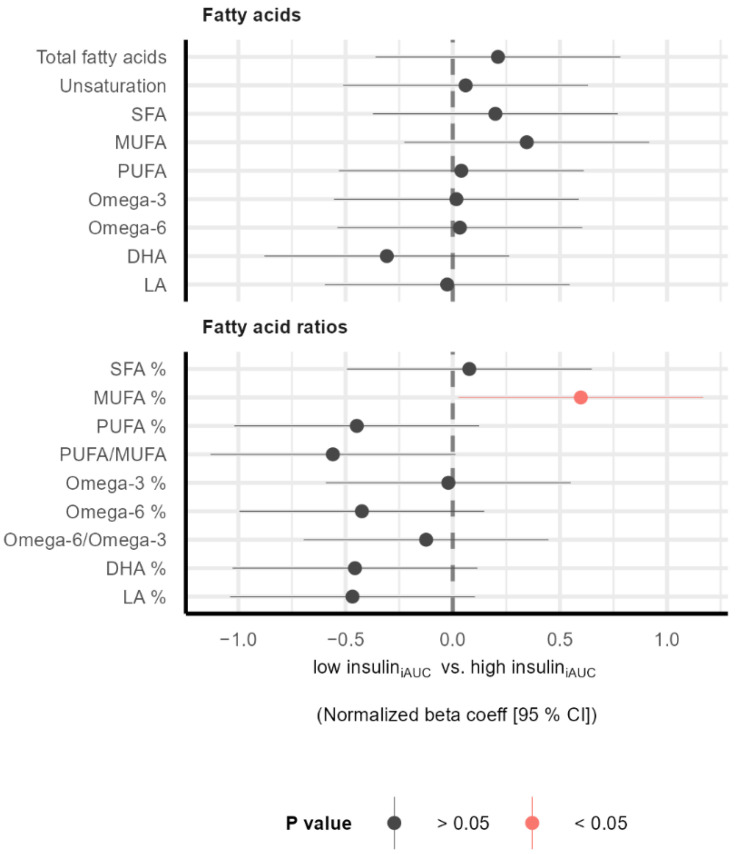
Fatty acids profile in plasma before intervention. The forest plot displays the normalized beta coefficients (mean difference) and 95% confidence interval for the difference between the low insulin_iAUC_ and high insulin_iAUC_groups. Estimates on the left and right side of the zero-line translate to lower and higher in the high insulin_iAUC_ group compared to the low insulin_iAUC_ group, respectively. Color denotes the *p* value. Abbreviations: DHA: Docosahexaenoic acid, DHA%: Ratio of docosahexaenoic acid to total fatty acids, iAUC: incremental Area Under the Curve, LA: Linoleic acid, LA%: Ratio of linoleic acid to total fatty acids, MUFA: Monounsaturated fatty acids, MUFA%: Ratio of monounsaturated fatty acids to total fatty acids, Omega-3%: Ratio of omega-3 fatty acids to total fatty acids, Omega-6%: Ratio of omega-6 fatty acids to total fatty acids, PUFA: Polyunsaturated fatty acids, PUFA%: Ratio of polyunsaturated fatty acids to total fatty acids, SFA: Saturated fatty acids, SFA%: Ratio of saturated fatty acids to total fatty acids, Unsaturation: Degree of unsaturation.

**Figure 2:**
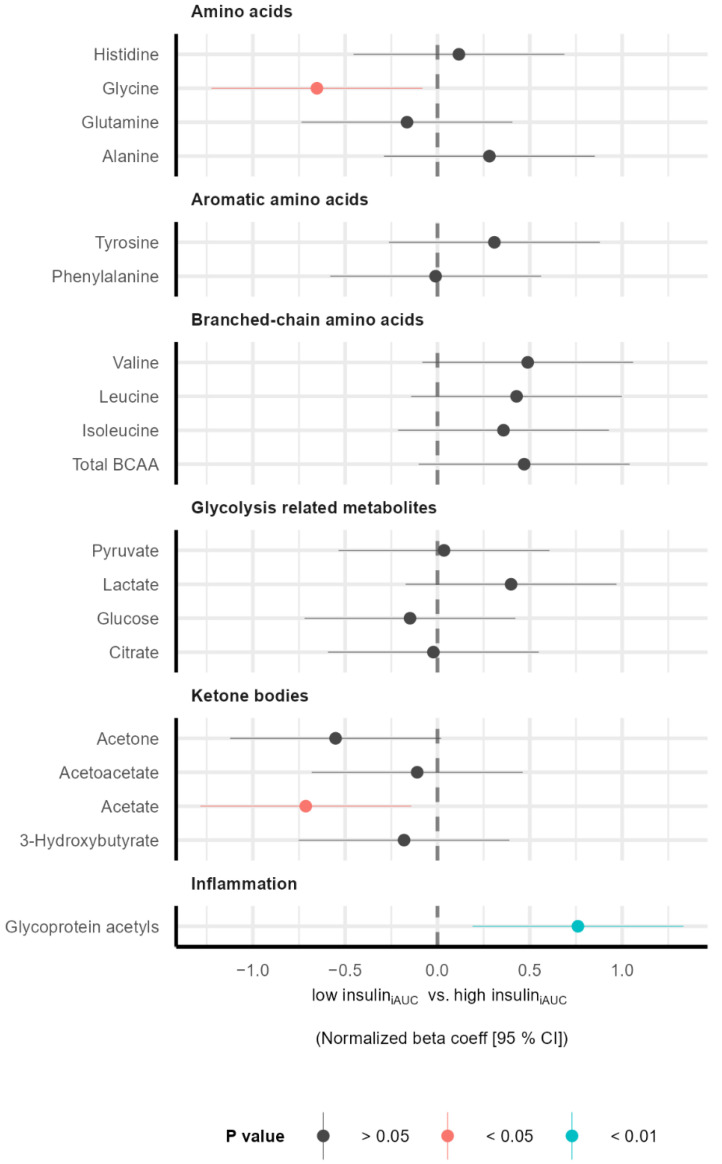
Amino acids, glycolysis-related metabolites, ketone bodies, and inflammation markers concentration in plasma before intervention. The forest plot displays the normalized beta coefficients (mean difference) and 95% confidence interval for the difference between the low insulin_iAUC_ and high insulin_iAUC_ groups. Estimates on the left and right side of the zero-line translate to lower and higher in the high insulin_iAUC_ group compared to the low insulin_iAUC_ group, respectively. Color denotes the *p* value. Abbreviations: BCAA: Branched-chain amino acids, iAUC: incremental Area Under the Curve.

To characterize individuals in the two subgroups (low insulin_iAUC_ and high insulin_iAUC_), we measured the plasma concentration of 250 metabolites. Compared to the low insulin_iAUC_ group, the high insulin_iAUC_ group had a higher ratio of MUFAs to total fatty acids (MUFA%) (Figure 1), lower levels of glycine and acetate, and a higher level of the inflammatory marker GlycA (Figure 2). Although there were no significant differences between the low insulin_iAUC_ and high insulin_iAUC_ groups regarding BCAAs, we observed a tendency to a higher individual (*p* = 0.09 for valine) and total BCAAs (*p* = 0.10) in the high insulin_iAUC_ group compared to the low insulin_iAUC_ group (Figure 2).

There were no differences in total cholesterol levels or lipid composition of lipoproteins between the low insulin_iAUC_ and high insulin_iAUC_ groups (Supplementary Figure S3) neither on the LP-IR score (data not shown). Despite this, we observed a tendency coherent with higher LP-IR score in the high insulin_iAUC_ compared to the low insulin_iAUC_ group: higher levels of large very low-density lipoproteins (VLDL) particles and lower levels of large HDL particles (Supplementary Figure S4). See Supplementary Table S3 for more information about all the NMR metabolites, fold-changes, *p* values, and adjusted *p* values.

### 3.3. Subgroup Differences in Gene Expression before Intervention

**Figure 3:**
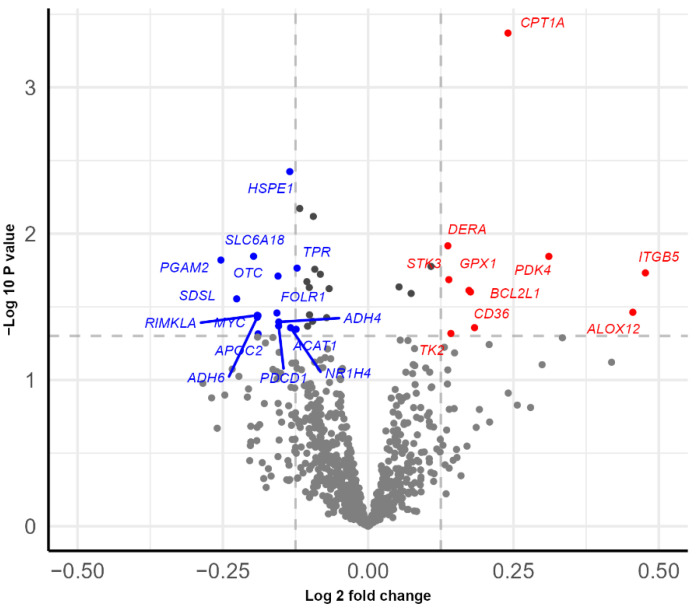
Volcano plot of differentially expressed genes before intervention. The linear model was adjusted by age, sex, BMI, and daily use of tobacco. Genes with *p* value < 0.05 (no FDR correction) and with an absolute fold change above 1.10 were highlighted. Genes on the left side of the volcano plot are down-regulated, and those on the right are up-regulated in the high insulin_iAUC_ group compared to the low insulin_iAUC_ group. Abbreviations: *ACAT1*: acetyl-CoA acetyltransferase 1, *ADH4*: alcohol dehydrogenase 4 (class II) pi polypeptide, *ADH6*: Alcohol dehydrogenase 6 (class V), *ALOX12*: arachidonate 12-lipoxygenase 12S type, *APOC2*: apolipoprotein C2, *BCL2L1*: BCL2 such as 1, *CD36*: CD36 molecule (alternatively *FAT*: Fatty acid traslocase, or *SCARB3*: Scavenger receptor class B member 3), *CPT1A*: carnitine palmitoyltransferase 1A, *DERA*: deoxyribose-phosphate aldolase, *FOLR1*: folate receptor alpha, *GPX1*: glutathione peroxidase 1, *HSPE1*: heat shock protein family E (Hsp10) member 1, iAUC: incremental Area Under the Curve, *ITGB5*: integrin subunit beta 5, *MYC*: MYC proto-oncogene, bHLH transcription factor, *NR1H4*: nuclear receptor subfamily 1 group H member 4 (alternatively *FXR*: Farnesol Receptor HRR-1), *OTC*: ornithine transcarbamylase, *PDCD1*: programmed cell death 1, *PDK4*: pyruvate dehydrogenase kinase 4, *PGAM2*: phosphoglycerate mutase 2, *RIMKLA*: ribosomal modification protein rimK such as family member A, *SDSL*: serine dehydratase like, *SLC6A18*: solute carrier family 6 member 18, *STK3*: serine/threonine kinase 3, *TK2*: thymidine kinase 2, *TPR*: translocated promoter region, nuclear basket protein.

We measured the gene expression of 778 gene transcripts involved in immunometabolism in PBMCs to investigate if the observed differences in insulin sensitivity and metabolites between the groups could be associated with differences in gene expression. We found 39 genes with a different expressions between the low insulin_iAUC_ and high insulin_iAUC_ groups at a nominal significance level of 0.05 (Figure 3). *CPT1A* (carnitine palmitoyl transferase 1A) was the most differentially expressed gene in terms of *p* value (*p* = 0.0004) and fold change (log2 fold-change = 0.24) and was higher expressed in the high insulin_iAUC_ group compared to the low insulin_iAUC_ group (Figure 3). However, none of the genes were significantly different between the groups after adjustment for false discovery rate (FDR < 0.1). See Supplementary Table S4 for more information about all the tested gene transcripts, fold-changes, *p* values, and adjusted *p* values.

### 3.4. Gene Set Enrichment Analysis and Competitive Gene Set Testing before Intervention

To understand patterns in gene expression between the low insulin_iAUC_ and high insulin_iAUC_ groups, we performed a gene enrichment analysis including the 39 genes with different expression (*p* < 0.05, without FDR correction) and using KEGG pathways and Gene Ontology (GO) terms. The top three KEGG pathways that showed enrichment in the high insulin_iAUC_ compared with the low insulin_iAUC_ group were thyroid cancer, ribosome, and fatty acid degradation (Table 2). In concordance, the top three GO terms (molecular function) were rRNA binding, large ribosomal subunit rRNA binding, and core promoter sequence-specific DNA binding. Furthermore, we performed a competitive gene set test (CAMERA) using the hallmark gene sets from MSigDB. Comparing the low insulin_iAUC_ and high insulin_iAUC,_ we found that the two most important altered gene sets were those related to MYC targets V1 (FDR = 0.0005) and MYC targets V2 (FDR = 0.02), which were both downregulated in the high insulin_iAUC_ group (Table 3).

### 3.5. Characterization of the Study Population in the Different Intervention Groups

We further investigated the effect of intervention with salmon fish protein on cardiometabolic risk markers in the low insulin_iAUC_ and high insulin_iAUC_ subgroups. Table 4 shows the anthropometric and biochemical characteristics of the study population allocated into distinct groups (low insulin_iAUC_ and high insulin_iAUC_) and treatments (placebo or fish protein) before the intervention. We observed the same pattern in glucose metabolism and insulin sensitivity as before subdividing into intervention groups: individuals in both low insulin_iAUC_ groups had lower glucose 2 h, fasting insulin, insulin 2 h, and HOMA-IR and higher Matsuda index, compared to individuals in the high insulin_iAUC_ groups (Table 4). We did not find significant differences in glycemic regulation (glucose, insulin, HOMA-IR, and HbA1c) after the intervention between individuals receiving a placebo or fish protein for the low insulin_iAUC_ or the high insulin_iAUC_ groups (data not shown).

### 3.6. Effect on Metabolic Profile after Fish Protein Intervention

**Figure 4:**
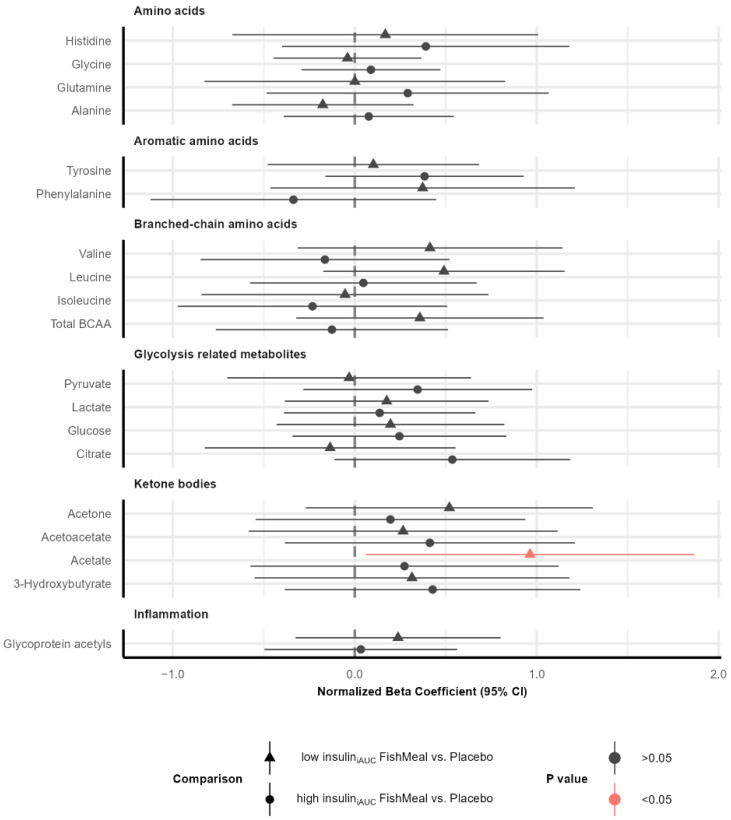
Changes in amino acids, glycolysis-related metabolites, ketone bodies, and inflammation markers concentration in plasma after the intervention. The forest plot displays the normalized beta coefficients (mean difference) and 95% confidence interval for the differences between the fish protein-treated low insulin_iAUC_ group and placebo-treated low insulin_iAUC_ group (triangles) and the differences between the fish protein-treated high insulin_iAUC_ group and placebo-treated high insulin_iAUC_ group (circles). Estimates on the left and right side of the zero-line translate to lower and higher in individuals that received the fish protein than those that received the placebo, respectively. Color denotes the *p* value. Abbreviations: BCAA: Branched-chain amino acids, iAUC: incremental Area Under the Curve.

We investigated the effect on plasma metabolites after 8-weeks of salmon fish protein intake compared to placebo in the low insulin_iAUC_ and high insulin_iAUC_ subgroups. We observed that fish protein-treated low insulin_iAUC_ participants had higher acetate levels than placebo-treated low insulin_iAUC_ participants (Figure 4). We did not find any other significant changes in plasma metabolites in the low insulin_iAUC_ or high insulin_iAUC_ groups after the intervention (Supplementary Figures S5–S7). See Supplementary Tables S5 and S6 for more information about all the NMR metabolites, fold-changes, *p* values, and adjusted *p* values.

### 3.7. Effect on Gene Expression after Fish Protein Intervention

**Figure 5:**
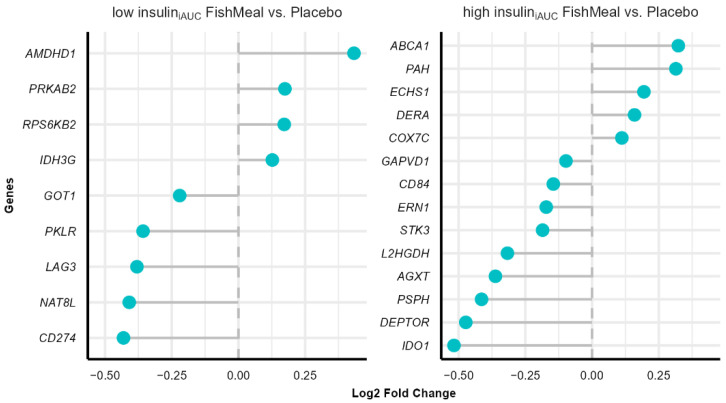
Differentially expressed genes in the low insulin_iAUC_ and high insulin_iAUC_ groups after the intervention. The figure shows the differentially expressed genes (*p* value < 0.05, no FDR correction) after the intervention in the low insulin_iAUC_ and high insulin_iAUC_ groups, sorted by fold change (blue dots). The genes with altered expression were obtained using a linear model adjusted for covariates (age, sex, BMI, and daily use of tobacco). Fold change represent the difference in PBMCs gene expression between fish protein-treated low insulin_iAUC_ and placebo-treated low insulin_iAUC_ group (**left panel**) and the difference between fish protein-treated high insulin_iAUC_ and placebo-treated high insulin_iAUC_ (**right panel**). Abbreviations: *ABCA1*: ATP binding cassette subfamily A member 1, *AGXT*: alanine-glyoxylate and serine-pyruvate aminotransferase, *AMDHD1*: amidohydrolase domain containing 1, *CD274*: CD274 molecule, *CD84*: CD84 molecule, *COX7C*: cytochrome c oxidase subunit 7C, *DEPTOR*: DEP domain containing MTOR interacting protein, *DERA*: deoxyribose-phosphate aldolase, *ECHS1*: enoyl-CoA hydratase, short chain 1, *ERN1*: endoplasmic reticulum to nucleus signaling 1, *GAPVD1*: GTPase activating protein and VPS9 domains 1, *GOT1*: glutamic-oxaloacetic transaminase 1, IDH3G: Isocitrate Dehydrogenase (NAD(+)) 3 Non-Catalytic Subunit Gamma, *IDO1*: indoleamine 2,3-dioxygenase 1, iAUC: incremental Area Under the Curve, *L2HGDH*: L-2-hydroxyglutarate dehydrogenase, *LAG3*: lymphocyte activating 3, *NAT8L*: N-acetyltransferase 8 like, *PAH*: phenylalanine hydroxylase, *PKLR*: pyruvate kinase L/R, *PRKAB2*: protein kinase AMP-activated non-catalytic subunit beta 2, *PSPH*: phosphoserine phosphatase, *RPS6KB2*: ribosomal protein S6 kinase B2, *STK3*: serine/threonine kinase 3.

Fold changes in gene expression were evaluated from before to after intervention with fish protein compared to placebo in the low insulin_iAUC_ and high insulin_iAUC_ groups. We found nine genes with altered expression in the fish protein-treated low insulin_iAUC_ group compared to the placebo-treated low insulin_iAUC_ group and 14 genes with altered expression in the fish protein-treated high insulin_iAUC_ group compared to placebo-treated high insulin_iAUC_ group, with a significance level below 0.05. After adjusting for FDR, none of the genes showed significant changes. Fold changes in gene expression during dietary interventions are typically not large, and in our study, the most altered genes in terms of fold change were *AMDHD1* (Amidohydrolase Domain-Containing Protein 1, log2 fold-change = 0.43) and *CD274* (CD274 molecule, log2 fold-change = −0.43) in the fish protein-treated low insulin_iAUC_ compared to placebo-treated low insulin_iAUC_ group. At the same time, *ABCA1* (ATP Binding Cassette Subfamily A Member 1, log2 fold-change = 0.32) and *IDO1* (Indoleamine 2,3-Dioxygenase 1, log2 fold-change = −0.51) were the most altered genes in the fish protein-treated high insulin_iAUC_ group compared to placebo-treated high insulin_iAUC_ group (Figure 5). We did not conduct gene enrichment analysis due to the small number of differentially expressed genes after the intervention. See Supplementary Tables S7 and S8 for more information about all the genes, fold changes, *p* values, and adjusted *p* values.

## 4. Discussion

In the present study, we investigated the metabolic and gene expression profile of subgroups of participants with different insulin responses to identify biomarkers of cardiometabolic risk and further determine whether any of these subgroups respond to an 8-week intervention with fish protein. Before the intervention, we found that the plasma level of GlycA and MUFAs, and the gene expression of *CPT1A* were higher, and glycine and acetate levels were lower in individuals in the high insulin_iAUC_ group compared to individuals in the low insulin_iAUC_ group. After the intervention, only acetate was increased in the low insulin_iAUC_ group treated with fish protein compared to the placebo-treated low insulin_iAUC_ group. However, none of the metabolites and gene transcripts before and after the intervention were significantly altered after adjustment for FDR, and hence the results should be carefully interpreted. 

We found that GlycA levels were higher in the high insulin_iAUC_ group, that is, the group with less insulin response (increased 2 h glucose, fasting insulin, 2 h insulin, and reduced Matsuda index), compared to the low insulin_iAUC_ group before intervention. GlycA is shown to be positively correlated with insulin resistance, BMI, markers of metabolic syndrome, and the ratio of leptin to adiponectin [45,46]. Furthermore, GlycA is associated with different inflammatory markers and is considered a biomarker of systemic inflammation and subclinical vascular inflammation [47]. Hence, GlycA is suggested as a new biomarker of cardiometabolic disease and T2D risk [19,22]. Although GlycA levels were higher in the high insulin_iAUC_ group compared to the low insulin_iAUC_ group, we did not observe any significant difference in hsCRP between the groups (Tables S1 and S4). The high insulin_iAUC_ group showed a tendency to a higher hsCRP concentration, and possibly we did not reach statistical significance due to a small sample size, intra-individual variations in hsCRP, or hsCRP did not capture the whole inflammatory process in our participants. This suggests that GlycA may act as a more precise risk marker of CVD and T2D compared to hsCRP [48]. Epidemiological studies supporting this hypothesis found that the association between GlycA and cardiovascular events, or T2D, was slightly attenuated by the addition of hsCRP to the regression model [48].

Before the intervention, we found that the high insulin_iAUC_ group had a higher level of MUFAs to total fatty acids ratio compared to the low insulin_iAUC_ group. In the NHANES study, the higher plasma concentrations of SFAs and MUFAs were associated with elevated HbA1c and fasting glucose levels [14], which is in line with our results. Furthermore, previous research has also shown that higher MUFA levels and lower PUFA and linolic acid levels are associated with increased cardiovascular risk in the FINNRISK study [13]. Hence, high circulating MUFA and low PUFA levels are linked to increased cardiovascular risk. 

*CPT1A* was upregulated in the high insulin_iAUC_ group compared to the low insulin_iAUC_ group before intervention and was one of the most differentially expressed genes in terms of the *p* value. The CPT1A protein, a key regulatory enzyme of β-oxidation, is in the outer membrane of the mitochondria. CPT1A facilitates the translocation of long-chain fatty acids across the mitochondrial membrane for fatty acid β-oxidation. Hence, our results may reflect a shift in metabolic sources from carbohydrates to fatty acids in the high insulin_iAUC_ group. In accordance with our observations, the Framingham Heart Study (FHS) Offspring Cohort found a positive association between mRNA expression of *CPT1A* in whole blood with fasting glucose and triglycerides, as well as BMI. Moreover, it was described that fat intake was negatively associated with *CPT1A* methylation, therefore, increasing *CPT1A* gene expression [49]. In an animal study, mitochondrial dysfunction caused by a high-fat diet was linked to insulin resistance in muscle, implying that excessive CPT1A activity overloads the mitochondria resulting from incomplete oxidation of long-chain fatty acids [50]. 

In our population, we found a tendency for a higher BCAAs plasma concentration in the high insulin_iAUC_ group compared to the low insulin_iAUC_ group before the intervention (Figure 2). We reached significance for valine and total BCAAs without age, sex, and BMI correction (data not shown). Both BCAAs and aromatic amino acids have been positively correlated with insulin resistance in T2D [51,52], and BCAAs are suggested as a metabolic signature associated with insulin resistance [53,54]. Increased levels of BCAAs may be a causal factor for developing insulin resistance and T2D by hampering insulin signaling pathways [55], and the accumulation of toxic BCAA metabolites triggers mitochondrial dysfunction [56]. 

Before the intervention, the individuals in the high insulin_iAUC_ group had lower levels of plasma glycine compared to the low insulin_iAUC_ group. In line with our results, most studies suggest an inverse association between glycine and prediabetes and T2D [51]. People with nondiabetic insulin resistance or impaired glucose tolerance have reduced circulating glycine [52]. It is unclear if reduced levels of glycine have an active role in the development of T2D, but it has been observed that interventions with delayed or reversed T2D (bariatric surgery or physical activity) are associated with an increase in circulating glycine concentrations [57,58,59]. Furthermore, glycine supplementation has been shown to give enhanced insulin response and glucose tolerance [60,61]. The consumption of 5.2 g of salmon protein (corresponding to 7.5 g fishmeal) was enough to significantly increase the post-prandial concentration of several amino acids, especially glycine [62]. However, in the present intervention study lasting for 8 weeks, we did not find an effect of fish protein on glucose metabolism, insulin response, or plasma glycine concentration in subgroups of individuals with increased T2D risk. Our fishmeal supplement provided 389 mg of glycine per day, which is considerably lower than the dosages used by others, including 1 mmol glycine/kg lean body mass (~5.63 g for a 75 kg patient) [60] and 5 g of glycine [61]. There are many metabolic effects of circulating glycine, and glycine supplementation may impact glucose tolerance [59]. Current evidence point to a glycine effect in the brain via dorsal vagal complex N-methyl-D-aspartate (NMDA) receptors, systemically reducing oxidative stress and inflammatory response and increasing insulin secretion in the islets via glycine receptors (GlyRs) [59]. 

Increased glucose levels promote reactive oxygen species (ROS) overproduction [63], resulting in morphological changes in mitochondria [64]. Excessive ROS could damage the proteins, lipids, and DNA in the mitochondria, leading to mitochondrial dysfunction and reduced mitochondria biogenesis [64]. Several studies have found a link between mitochondrial dysfunction and insulin resistance in various tissues [64]. We found a down-regulation in the Myc targets V1 and V2 pathways in the high insulin_iAUC_ group (Table 3), which could be a sign of reduced mitochondrial biogenesis, as Myc induces mitochondrial biogenesis and increases mitochondrial function through many pathways [65]. Moreover, alterations in the oxidizing environment of the endoplasmic reticulum (ER) can induce ER stress. There is evidence that beta-cell ER stress in patients with T2D can cause beta-cell dysfunction by decreasing insulin synthesis and secretion [66]. This could be a reason for the ribosome-enriched pathway found in the high insulin_iAUC_ group before intervention (Table 2). 

We observed lower concentrations of acetate in the high insulin_iAUC_ compared to the low insulin_iAUC_ group before intervention (Figure 2). In contrast, acetate was associated with prediabetes and T2D in other studies [51]. Acetate is a short-chain fatty acid produced by microbiota. The microbiome is a potential source of biologically active metabolites, frequently linked with diet composition [67]. It has been shown in healthy subjects that prebiotics increased plasma glucagon-like peptide 1 (GLP-1) and peptide YY (PYY) concentrations, whereas postprandial plasma glucose response decreased after a standardized meal [68]. In another RCT, healthy individuals treated with long-chain MUFAs derived from fish oil increased GLP-1 secretion. The proposed mechanism is linked to gut microbiota producing short-chain fatty acids that act through the G protein-coupled receptors expressed on enteroendocrine cells, enteric neurons, and enteric leukocytes [69]. In our study, acetate was increased in the low insulin_iAUC_ group after fish protein intervention compared to placebo treatment, while no change was observed in the high insulin_iAUC_ group. Recent studies show that gut microbial composition may help to identify individuals who may benefit from dietary interventions [70].

This study has several strengths and limitations. One strength is the detailed profiling of both metabolites and gene expressions in subgroups of people with increased T2D risk. The small number of participants in the phenotypic subgroups in the intervention is a limitation of the study, giving a higher risk of both false positive and false negative findings. The results from small studies cannot be generalized to the population as a whole. Furthermore, the inclusion criteria for participation were increased risk of T2D, and hence the lack of a healthy control group is a limitation of the present study. Lastly, the short-term duration of the present study does not reflect the long-term effects. Despite this, we were able to identify metabolic biomarkers (GlycA, MUFA %, glycine, and acetate), as well as candidate gene expression patterns in the group of participants that displayed high insulin_iAUC_.

## 5. Conclusions

In conclusion, our results support that several plasma metabolites (GlycA, MUFA %, glycine, and acetate) may serve as biomarkers to predict the progression of T2D; however, the usefulness of these needs further testing and validation in prospective studies. Whether these newly suggested biomarkers are more sensitive to dietary interventions in people with increased risk of T2D is still to be elucidated.

Results from this and similar intervention studies are important for hypothesis generation in terms of pathophysiology and clinical manifestation in subgroups of individuals in heterogeneous populations.

## Figures and Tables

**Table 1:** Anthropometric and biochemical characteristics of subgroups before intervention.

Variable	Low Insulin_iauc_	High Insulin_iauc_	*p* Value
	*n* = 24	*n* = 24	
Sex (*n* female, %)	17 (71%)	15 (63%)	
Tobacco use daily (*n*, %)	2 (9.1%)	5 (26.3%)	
Age (y)	53.0 (46.3–64.0)	61.5 (49.5–65.0)	n.s
Weight (kg)	94.9 ± 16.8	100.3 ± 19.0	n.s
BMI (kg m^−2^)	32.4 (28.9–35.1)	33.0 (31.0–36.4)	n.s
f. Glucose (mmol L^−1^)	5.4 (5.0–5.8)	5.2 (5.1–5.7)	n.s
Glucose 2 h (mmol L^−1^)	4.7 ± 1.1	6.9 ± 1.1	***
HbA1c (%)	5.8 ± 0.3	5.9 ± 0.3	n.s
f. Insulin (pmol L^−1^)	56 (44–92)	124 (102–196)	***
Insulin 2 h (pmol L^−1^)	154 (120–225)	804 (661–1152)	***
Insulin iAUC (pmol h L^−1^)	113 (60–163)	766 (647–1058)	***
HOMA-IR	2.4 (1.7–3.7)	4.9 (3.7–5.8)	***
Matsuda index	6.9 (4.9–10.7)	1.8 (1.1–2.1)	***
Triglycerides (mmol L^−1^)	1.4 ± 0.7	1.6 ± 0.6	n.s
Total cholesterol (mmol L^−1^)	5.3 ± 1.3	5.0 ± 0.8	n.s
HDL-C (mmol L^−1^)	1.4 (1.2–1.8)	1.2 (1.1–1.4)	n.s
LDL-C (mmol L^−1^)	3.5 (2.6–4.3)	3.3 (2.9–4.2)	n.s
ApoA1 (g L^−1^)	1.7 (1.5–1.8)	1.5 (1.4–1.7)	n.s
Apo B (g L^−1^)	1.1 ± 0.3	1.0 ± 0.2	n.s
hsCRP (mg L^−1^)	3.4 (2.1–4.6)	4.2 (2.6–7.3)	n.s
Systolic BP (mm Hg)	117 ± 13	123 ± 17	n.s
Diastolic BP (mm Hg)	69 (58–78)	71 (66–78)	n.s

*** *p* < 0.001 low insulin_iAUC_ vs. high insulin_iAUC_. Parametric data are expressed as mean ± SD, and non-parametric data as median and interquartile range. Differences between tertiles were assessed using an ANOVA with Tukey posthoc test for parametric variables or Kruskal-Wallis rank sum test with Pairwise Wilcoxon Rank Sum Tests for non-parametric variables. Abbreviations: ApoA1: apolipoprotein A-I, Apo B: apolipoprotein B, BMI: body mass index, BP: blood pressure, f.: fasting, HbA1c: hemoglobin A1c, HDL-C: high-density lipoprotein cholesterol, HOMA-IR: homeostatic model assessment for insulin resistance, hsCRP: high sensitivity C-reactive protein, iAUC: incremental Area Under the Curve, LDL-C: low-density lipoprotein cholesterol, n.s: not significant.

**Table 2:** Enriched KEGG pathways and top 5 GO terms before intervention.

	Pathway Name	Ratio	*p* Value	Genes Symbol
KEGG Pathway ID				
hsa05216	Thyroid cancer	4/12	2.00 × 10^−3^	MYC, MAPK1, TP53, TPR
hsa03010	Ribosome	2/2	2.45 × 10^−3^	RPLP0, RPL23
hsa00071	Fatty acid degradation	4/17	8.02 × 10^−3^	ACAT1, ADH4, ADH6, CPT1A
hsa05219	Bladder cancer	3/14	2.89 × 10^−2^	MYC, MAPK1, TP53
GO term ID				
GO:0019843	rRNA binding	3/3	1.17 × 10^−4^	NPM1, RPLP0, RPL23
GO:0070180	large ribosomal subunit rRNA binding	2/2	2.45 × 10^−3^	RPLP0, RPL23
GO:0001046	core promoter sequence-specific DNA binding	3/7	3.55 × 10^−3^	MYC, NPM1, TP53
GO:0003735	structural constituent of ribosome	2/3	7.12 × 10^−3^	RPLP0, RPL23
GO:0003723	RNA binding	7/50	9.25 × 10^−3^	HSPE1, IMPDH2, NPM1, RPLP0, TP53, TPR, RPL23

For KEGG pathways and GO terms (molecular function), we performed a gene enrichment analysis using the list of 39 differentially expressed genes (*p* value < 0.05, no FDR correction) obtained with the adjusted linear model as input. All the analyses were performed using the genes in the nCounter Human Metabolic Pathways Panel plus 30 additional genes from the custom panel (Supplementary Tables S1 and S2). The displayed pathways are differentially expressed in the high insulin_iAUC_ group compared to the low insulin_iAUC_ group. Pathways are sorted by *p* value. The ratio column represents the number of genes with differential expression in that set and the total number of genes in that pathway. The symbols of the differentially expressed genes in each pathway are shown.

**Table 3:** Top 10 differentially expressed hallmark gene sets (MSigDB) before intervention.

Hallmark (MSigDB) Gene Sets	Number of Genes in the SET	Direction	*p* Value	FDR
MYC targets V1	22	Down	1.18 × 10^−5^	5.92 × 10^−4^
MYC targets V2	6	Down	9.73 × 10^−4^	2.43 × 10^−2^
IL6 JAK STAT3 signaling	19	Up	3.15 × 10^−3^	5.05 × 10^−2^
Epithelial mesenchymal transition	11	Up	5.12 × 10^−3^	5.05 × 10^−2^
TGF beta signaling	4	Up	5.63 × 10^−3^	5.05 × 10^−2^
Wnt beta catenin signaling	4	Down	6.05 × 10^−3^	5.05 × 10^−2^
TNFA signaling via NFKB	31	Up	1.55 × 10^−2^	1.11 × 10^−1^
Apoptosis	21	Up	2.63 × 10^−2^	1.64 × 10^−1^
Protein secretion	6	Up	3.26 × 10^−2^	1.69 × 10^−1^
Interferon gamma response	28	Up	3.37 × 10^−2^	1.69 × 10^−1^
Angiogenesis	2	Up	4.46 × 10^−2^	2.03 × 10^−1^

For hallmark gene sets, we performed a competitive gene set test named CAMERA. The analysis was performed using the genes in the nCounter Human Metabolic Pathways Panel plus 30 additional genes from the custom panel (Supplementary Tables S1 and S2). The displayed gene sets are differentially expressed in the high insulin_iAUC_ group compared to the low insulin_iAUC_ group. Gene sets are sorted by *p* value. The Direction column indicates if the gene set is down- or up-regulated in the high insulin_iAUC_ group compared to the low insulin_iAUC_ group. Abbreviations: FDR: False Discovery Rate, IL6: Interleukin-6, JAK: Janus Kinase, MSigDB: Molecular Signature Database, MYC: Myc proto-oncogene bHLH transcription factor, NFKB: Nuclear factor-kappa B, STAT3: Signal Transducer and Activator of Transcription 3, TGF beta: Transforming Growth Factor Beta, TNFA: Tumor Necrosis Factor-Alpha.

**Table 4:** Anthropometric and biochemical characteristics of the study population in the different intervention groups.

	Low Insulin_iauc_ Placebo	Low Insulin_iauc_ Fish Protein	High Insulin_iauc_ Placebo	High Insulin_iAUC_ Fish Protein	*p* Value
	*n* = 8	*n* = 16	*n* = 12	*n* = 12	
Sex (*n* female, %)	5 (62%)	12 (75%)	9 (75%)	6 (50%)	
Tobacco use daily (*n*, %)	2 (25%)	0 (0%)	2 (10%)	3 (25%)	
Age (y)	56.9 ± 13.9	52.1 ± 10.6	59.0 ± 9.2	56.1 ± 10.6	n.s
Weight (kg)	92.9 ± 19.6	95.9 ± 15.8	97.0 ± 13.9	104.0 ± 23.3	n.s
BMI (kg/m^2^)	30.2 (28.5–34.1)	32.8 (29.4–35.7)	34.1 (31.2–36.0)	32.9 (30.8–37.6)	n.s
f. Glucose (mmol L^−1^)	5.8 (5.0–6.2)	5.2 (5.0–5.6)	5.3 (5.0–5.7)	5.2 (5.1–5.8)	n.s
Glucose 2 h (mmol L^−1^)	4.9 ± 1.1	4.7 ± 0.4	6.8 ± 1.4	6.9 ± 0.8	**, ###
HbA1c (%)	5.9 (5.7–6.1)	5.8 (5.7–5.9)	5.7 (5.6–5.8)	5.9 (5.7–6.3)	n.s
f. Insulin (pmol L^−1^)	73 ± 65	66 ± 61	139 ± 70	147 ± 62	**, ###
Insulin 2 h (pmol L^−1^)	148 (133–228)	164 (115–218)	764 (685–1152)	861 (661–1171)	***, ###
Insulin iAUC (pmol h L^−1^)	116 (73–163)	113 (53–159)	766 (647–1058)	830 (638–1078)	***, ###
HOMA-IR	2.2 (1.6–3.2)	2.4 (1.8–3.7)	4.3 (3.4–8.6)	5.1 (4.4–7.6)	###
Matsuda index	6.2 (4.6–11.2)	6.8 (5.1–8.6)	1.8 (1.2–2.2)	1.7 (1.1–2.0)	***, ###
Triglycerides (mmol L^−1^)	1.2 ± 0.4	1.5 ± 0.4	1.6 ± 0.7	1.6 ± 0.4	n.s
Total cholesterol (mmol L^−1^)	5.0 ± 0.8	5.4 ± 1.6	4.9 ± 0.8	5.2 ± 0.8	n.s
HDL-C (mmol L^−1^)	1.5 (1.2–1.8)	1.4 (1.2–1.8)	1.3 (1.1–1.5)	1.2 (1.0–1.3)	n.s
LDL-C (mmol L^−1^)	3.2 ± 0.6	3.7 ± 1.0	3.1 ± 0.2	3.8 ± 1.0	n.s
ApoA1 (gL^−1^)	1.8 (1.5–1.8)	1.7 (1.5–1.8)	1.6 (1.4–1.7)	1.5 (1.4–1.6)	n.s
ApoB (gL^−1^)	0.9 ± 0.2	1.1 ± 0.2	1.0 ± 0.2	1.1 ± 0.2	n.s
hsCRP (mg L^−1^)	4.0 ± 2.4	3.5 ± 3.1	4.6 ± 3.0	5.4 ± 3.1	n.s
Systolic BP (mm Hg)	119 ± 16	116 ± 13	124 ± 20	122 ± 13	n.s
Diastolic BP (mm Hg)	68 ± 13	70 ± 11	70 ± 8	71 ± 11	n.s

** *p* < 0.01 and *** *p* < 0.001 low insulin_iAUC_ placebo vs. high insulin_iAUC_ placebo. ### *p* < 0.001 low insulin_iAUC_ fish protein vs. high insulin_iAUC_ fish protein. Parametric data are expressed as mean ± SD, and non-parametric data as median and interquartile range. Differences between groups were assessed using an ANOVA with Tukey posthoc test for parametric variables or Kruskal-Wallis rank sum test with Pairwise Wilcoxon Rank Sum Tests for non-parametric variables. Abbreviations: ApoA1: apolipoprotein A-I, Apo B: apolipoprotein B, BMI: body mass index, BP: blood pressure, f: fasting, HbA1c: hemoglobin A1c, HDL-C: high-density lipoprotein cholesterol, HOMA-IR: homeostatic model assessment for insulin resistance, hsCRP: high sensitivity C-reactive protein, iAUC: incremental Area Under the Curve, LDL-C: low-density lipoprotein cholesterol, n.s: not significant.

## Data Availability

The datasets used are available from the corresponding author upon reasonable request.

## Supplementary Materials

The following supporting information can be downloaded at: https://www.mdpi.com/article/10.3390/nu14235165/s1, Figure S1: Methods flow chart before intervention; Figure S2: Methods flow chart after the intervention; Figure S3: Cholesterol concentration, lipoprotein sizes, and lipid composition and apolipoprotein concentration in plasma before intervention; Figure S4: Lipoprotein subclasses concentration and composition in plasma before intervention; Figure S5: Changes in fatty acid profile in plasma after the intervention; Figure S6: Changes in cholesterol concentration, lipoprotein size and lipid composition and apolipoprotein concentration in plasma after the intervention; Figure S7: Changes in lipoprotein subclass concentration and composition in plasma after the intervention. Table S1: Nanostring nCounter Metabolic Pathway Panel; Table S2: Additional code-sets covering immune and inflammation response and lipid metabolism-related mRNAs; Table S3: Metabolomic analysis before the intervention. Low insulin_iAUC_ vs. high insulin_iAUC_ group; Table S4: Gene expression analysis before the intervention. Low insulin_iAUC_ vs. high insulin_iAUC_ group; Table S5: Metabolomic analysis after the intervention. Fish protein-treated low insulin_iAUC_ vs. placebo-treated low insulin_iAUC_; Table S6: Metabolomic analysis after the intervention. Fish-protein-treated high insulin_iAUC_ vs. placebo-treated high insulin_iAUC_; Table S7: Gene expression after the intervention. Fish-protein-treated low insulin_iAUC_ vs. placebo-treated low insulin_iAUC_; Table S8: Gene expression analysis after the intervention. Fish-protein-treated high insulin_iAUC_ vs. placebo-treated high insulin_iAUC_.
